# Two New Cases of Pulmonary Infection by *Mycobacterium shigaense*, Japan

**DOI:** 10.3201/eid2611.200315

**Published:** 2020-11

**Authors:** Shiomi Yoshida, Tomotada Iwamoto, Takehiko Kobayashi, Ryohei Nomoto, Yoshikazu Inoue, Kazunari Tsuyuguchi, Katsuhiro Suzuki

**Affiliations:** National Hospital Organization Kinki-chuo Chest Medical Center, Sakai, Osaka, Japan (S. Yoshida, T. Kobayashi, Y. Inoue, K. Tsuyuguchi, K. Suzuki);; Kobe Institute of Health, Kobe, Japan (T. Iwamoto, R. Nomoto)

**Keywords:** Mycobacterium shigaense, bacteria, tuberculosis and other mycobacteria, nontuberculous mycobacteria, respiratory infections, infection, pulmonary disease, fibrocavitary, nodular bronchiectasis, whole-genome sequencing, comparative genomics, virulence, Japan

## Abstract

We report 2 case-patients in Japan with *Mycobacterium shigaense* pulmonary infections. One patient was given aggressive treatment and the other conservative treatment, according to distinctive radiologic evidence. A close phylogenetic relationship based on whole-genome sequencing was found between strain from the conservatively treated patient and a reference strain of cutaneous origin.

Nontuberculous mycobacteria (NTM) are ubiquitous organisms whose pathogenicity might vary according to the immune status of the host (*1*). An increase in incidence of pulmonary NTM infections among immunocompetent patients in recent years is an emerging public health concern (*2*).

The most predominant pulmonary *Mycobacterium avium–intracellulare* complex (MAC) disease has 2 possible radiologic patterns: a fibrocavitary (FC) type, which results in progressive radiographic abnormalities associated with a difficult-to-treat outcome; and a nodular bronchiectasis (NB) type, which is stable, often associated with chronic bronchiectasis (BE). International guidelines suggest slightly different multidrug treatment regimens for these types: conservative nonchemical treatment or intermittent oral therapy for NB-type and daily treatment for FC-type (*3*). Clinical and radiographic features of pulmonary disease caused by infection with rare NTM resemble those of MAC.

*M. shigaense* is a unique species of the *M. simiae* complex reported in 2012 (*4*). This slow-growing mycobacteria, UN-152^T^ (JCM 32072^T^ and DSM 46748^T^), caused cutaneous disease in an immunosuppressed patient (*4*). Since then, 5 cases of patients with *M. shigaense* infection have been reported, in the form of skin or disseminated diseases associated with cellular immunodeficiency (*5*). In 2014, another case of *M. shigaense* infection was reported in a respiratory sample of a patient in Japan (*6*). To date, *M. shigaense* has been found in eastern Asia, China, and Japan (*5*,*6*), but its transmission routes and sources have not been identified.

We isolated *M. shigaense* from 2 patients, 1 with FC-type disease and 1 with NB-type disease. We summarize clinical features and drug regimens (Appendix) for these patients and describe genomic comparison of strains associated with different treatments for infection with *M. shigaense*.

## The Study

This retrospective study was approved by the Institutional Review Board of the Kinki-Chuo Chest Medical Center (approval code 689) and has been performed in accordance with the ethical standards in the 1964 Declaration of Helsinki and its later amendments or comparable ethical standards. We required that all patients provide written informed consent before information was collected.

Case-patient 1 was an 88-year-old HIV-negative man admitted to our hospital in 2018. He had complications from stable interstitial pneumonia and an increased productive cough and fever. Chest radiograph and transverse computed tomography showed left-side consolidation.

**Figure 1:**
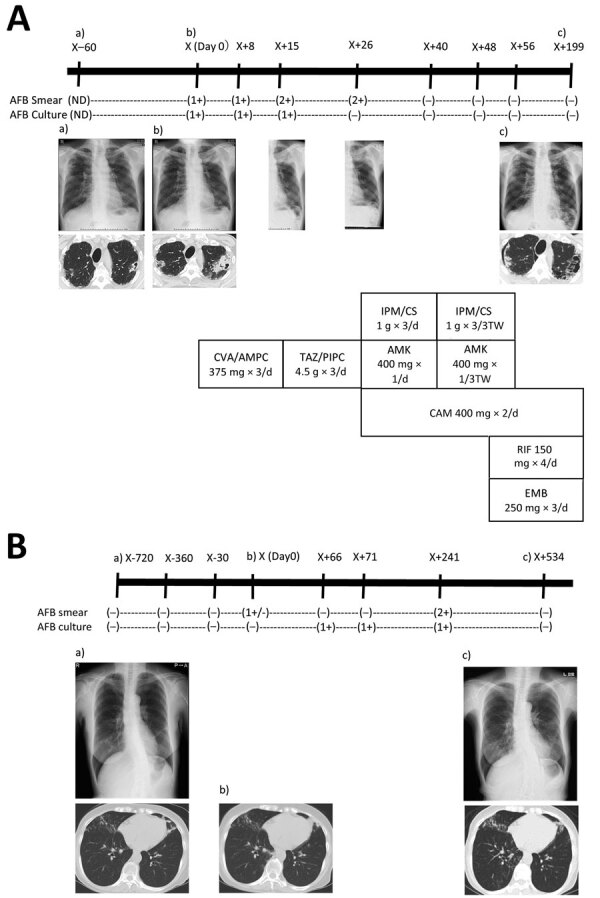
Radiographic and therapeutic drug monitoring for 2 patients with pulmonary disease caused by *Mycobacterium shigaense*. Each panel shows the timeline at the top (X, initial hospitalization period for *M. shigaense* disease) with smear results and chest radiograph (top) and chest CT (bottom) images below. The chemotherapy regimen is provided (Appendix). A) Case 1, patient with FC-type disease. a) Chest radiograph shows abnormal nodular shadows and a small calcification in the right upper and middle lung fields on day 60 before initial hospitalization. b) Chest radiograph taken 2 months later showed a more indistinct bilateral contour of the lung; there was increased consolidation of a cavitary lesion in the right upper lobe and a centrilobular nodule with branching in the left upper lobe on transverse chest CT. Lesions including progressive cavities are shown in the right upper and middle lung fields. c) Chest CT shows reduction in cavities and consolidation in the left lobe on day 199. B) Case 2, patient with NB-type disease. a) Chest CT showed a small nodular shadow in the right lower lung field. Image showed bronchiectasis in the left middle lobe and the lingular segment of the right upper lobe. There was peribronchiectasic consolidation and multiple small nodules suggesting bronchiolitis in both lungs. b) After 24 months, chest CT showed a stable extent of scattered small nodules including bronchiectasis just beneath the pleura and pleural thickening in the right middle lobe. c) Chest CT showed bronchiectasis in the right middle lobe. According to the number of AFB seen by Ziehl-Neelsen method for acid-fast staining, smear results were classified as 3+, 2+, 1+, or ±. –, negative; +, positive. AFB, acid-fast bacilli; AFB culture result –, culture negative; +, culture positive. AMK, amikacin; CAM, clarithromycin; CVA/AMPC, clavulanic acid/amoxicillin; CT, computed tomography; EMB, ethambutol; FC, fibrocavitary; IPM/CS, imipenem/cilastatin; NB, nodular bronchiectasis; ND, no data; RIF, rifampin; TAZ/PIPC, tazobactam/piperacillin.

Two months later, sputum cultures were positive for *M. shigaense*, identified by partial DNA sequences of the 16S rRNA, *hsp65*, and *rpoB* genes. Thereafter, multidrug treatment was orally administered for 12 months. After »1 month of treatment, his sputum was culture negative and negative for acid-fast bacilli (AFB). The patient showed clinical improvement and decreased symptoms. He has remained culture negative for >1 year (Figure 1, panel A).

Case-patient 2 was a 78-year-old HIV-negative woman referred for an evaluation of previously diagnosed chronic BE since 2016. She had an NB-type radiologic pattern. AFB smear and culture test results were negative on 3 consecutive sputum samples.

This patient was not initially given antimicrobial drugs and was evaluated by expectorated sputum examinations at follow-up. After 2 years, *M. shigaense* was isolated from subsequent sputum samples on 3 occasions. Because there were no respiratory symptoms, the patient was not initially given antimicrobial drugs after diagnosis. Spontaneous culture conversion was found after 3 consecutive negative sputum cultures, and the negative status was maintained during the follow-up period of >1 year (Figure 1, panel B).

We performed drug susceptibility testing considering MAC breakpoints and using broth microdilution according to Clinical and Laboratory Standards Institute guidelines (*7*). Testing showed that isolates of both patients were susceptible to clarithromycin, amikacin, moxifloxacin, and linezolid.

Whole-genome sequencing was performed on an initial isolate from each case-patient (strain KC8 from case-patient 1 and strain KC354 from case-patient 2). DNA sequence libraries were prepared by using the QIAseq FX DNA Library Kit (QIAGEN, https://www.qiagen.com) using 50 ng *M. tuberculosis* genomic DNA, followed by paired-end sequencing using Illumina MiSeq Reagent Kit version 3 (600 cycles) (Illumina, https://www.illumina.com).

We conducted average nucleotide identity basic local sequence alignment tool analysis by using JSpecies version 1.2.1 (*8*). Average nucleotide identity values for strains KC8 and KC354 clearly indicated that these 2 patients were infected by *M. shigaense* (Table 1). Identification of single-nucleotide polymorphisms (SNPs) in these isolates, compared with those in *M. shigaense* JCM 32072^T^, was conducted by using the BactSNP pipeline (*9*). KC354 had 19 SNPs, whereas KC8 had 6,826 SNPs (Table 2).

**Table 1:** Nucleotide identities of *Mycobacterium species* calculated by using average nucleotide identity BLAST ANI analysis*

Strain	Case 2, NB-type, KC354	Case 1, FC-type, KC8	*M. shigaense* JCM 32072^T^†	*M. shigaense* SCY‡	*M. triplex* DSM 4426^T^	*M. simiae* DSM 44165^T^
Case 2, NB-type, KC354	100.00	99.72	99.98	98.79	85.16	84.49
Case 1, FC-type, KC8	99.72	100.00	99.97	99.76	85.16	84.48
*M. shigaense* JCM 32072^T^†	99.98	99.97	100.00	99.81	85.17	84.50
*M. shigaense* SCY‡	99.79	99.76	99.81	100.00	85.18	84.49
*M. triplex* DSM 44626^T^	85.16	85.16	85.17	85.18	100.00	85.31
*M. simiae* DSM 44165^T^	84.49	84.48	84.50	84.19	85.31	100.00

*Values are percentages. ANI, average nucleotide identity; FC, fibrocavitary; NB, nodular bronchiectasis.  †*M. shigaense* JCM 32072^T^ was obtained from a skin biopsy specimen of a Japanese man ‡*M. shigaense* SCY was isolated from a skin biopsy specimen of a Chinese woman.

**Table 2:** SNP detection using *Mycobacterium shigaense* JCM 32072^T^ as reference genome for 2 strains isolated*

Strain no.	No. SNPs	Mapped region to reference genome†		Pseudogenome by BactSNP, bases
		Length, bases	Coverage ratio	
Case 2, NB-type, KC354	19	5,232,622	0.99999	5,182,569
Case 1, FC-type, KC8	6,826	5,200,032	0.99376	5,138,016

*SNPs called by BactSNP pipeline (*9*). FC, fibrocavitary; NB, nodular bronchiectasis; SNP, single-nucleotide polymorphism. †Coverage depth is >5 at each position of the reference genome (5,232,660).

**Figure 2:**
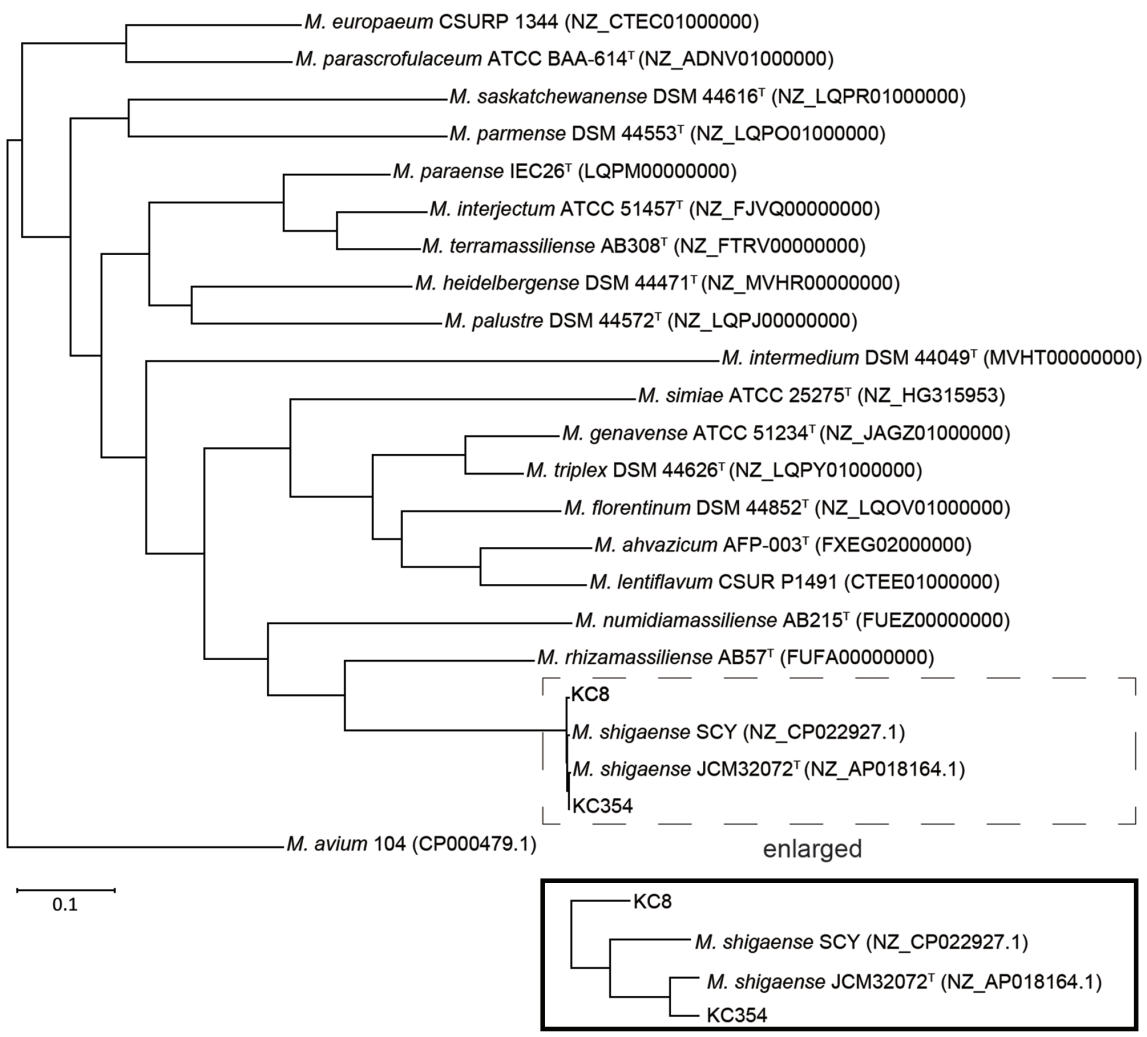
Phylogenetic tree based on whole-genome sequence data of 19 species in the *Mycobacterium simiae* complex and *M. avium* 104 from the GenBank database. The tree was constructed by using concatenated alignments of the 1,399 core genes with Roary, the pan genome pipeline (https://sanger-pathogens.github.io/Roary), and displayed by using Dendroscope (https://www.dendroscope.org). Box at the bottom shows an enlarged version of the branch of *M. shigaense* in the tree. Scale bar indicates nucleotide substitutions per site.

A core genes phylogenetic tree for genome sequences of 19 *M. simiae* complex and *M. avium* 104 was reconstructed by using Roary version 3.11.2 (*10*) (Figure 2). *M. shigaense* strains were most closely related to *M. rhizamassiliense* (*11*); KC8 was less related to *M. shigaense* JCM 32072^T^ than to *M. shigaense* SCY. Raw sequence reads of *M. shigaense* KC8 and *M. shigaense* KC354 were deposited in the DNA Data Bank of Japan Sequence Read Archive (DNA Sequence Read Archive, https://www.ddbj.nig.ac.jp/dra/index.html) under study accession no. DRA009490.

## Conclusions

NB-type infection with *M. shigaense* is considered sufficiently indolent that careful longitudinal appraisal without therapy is safe and poses little risk for rapid progression (*12*,*13*). The previously reported case-patient with NB-type pulmonary *M. shigaense* disease did not have a history of immunosuppressive therapy or clinical symptoms but was treated successfully (*6*). Clinical data, including the presence or absence of underlying BE as a concurrent condition, was insufficient (*6*). Our NB-type patient with chronic BE was considered not to have a clinically serious condition and required no treatment. In contrast, the patient with progressive FC-type infection was given chemotherapy that resulted in improvement observed by computed tomography (Figure 1). We showed that clinical features of *M. shigaense* disease resemble those of MAC disease, but radiographic differences indicated that MAC disease was more serious.

We also observed genomic diversity of *M. shigaense*. Our comparative genomic analysis showed that strain KC354 obtained from the NB-type patient was closely related to *M. shigaense* JCM 32071^T^. In contrast, strain KC8 obtained from the FC-type patient showed a large number of SNPs when compared with the type strain. This result might explain the increased virulence of strain KC8.

More than 50% of stable pulmonary MAC disease patients have spontaneous sputum conversion without treatment (*3*,*14*). Bacterial genotypic comparison between patients with spontaneous sputum conversion and those with serial sputum-positive cultures might identify patients who are likely to profit from antimicrobial drug therapy. Our next-generation sequencing findings indicated that differences in the genetic background of the pathogens might aid physicians with clinical decisions regarding therapy initiation. We believe that accumulation of genomic data for clinical strains should be helpful for future comparative studies and will probably lead to diagnosis of more cases. Further studies of this relatively new pathogen with larger sample sets are needed to identify clear, reliable, and clinical markers that predict the virulence of *M. shigaense*.

Current treatment for pulmonary mycobacterial disease recommends ethambutol, rifampin, and macrolides (*1*,*13*). Little is known regarding response of antimicrobial agents and clinical outcome for this rare species. Therefore, a better understanding of drug susceptibility of the pathogen is necessary to provide suitable treatment. Most patients reported with *M. shigaense* disease were given multiple agents: 4 patients showed improvement after receiving a clarithromycin-based treatment for 4–12 months (no data were available for linezolid) (*6*,*15*). In comparison to previous cases of *M. shigaense* disease (*5*), for which the pathogen was susceptible to clarithromycin and moxifloxacin, our isolates showed susceptibility to amikacin. We believe that a synergistic response of antimicrobial drugs against *M. shigaense* requires further evaluation.

In summary, we found that the NB-type *M. shigaense* pulmonary strain was closely related to the cutaneous reference strain, but the more pathogenic FC-type strain differed considerably. Our results for this rare species open possibilities for further investigation into this neglected NTM disease and provide indications for the need for therapy.

Appendix Additional information on 2 new cases of pulmonary infection by *Mycobacterium shigaense*, Japan.
