# Supplementary material for: Two New Cases of Pulmonary Infection by Mycobacterium shigaense, Japan
Source: Emerg Infect Dis. 2020 Nov;26(11):2728–32. doi: 10.3201/eid2611.200315 (PMC7588508; doi:10.3201/eid2611.200315)
Supplement: Appendix — Additional information on 2 new cases of pulmonary infection by Mycobacterium shigaense, Japan. [file 20-0315-Techapp-s1.pdf]

Article DOI: https://10.3201/eid2611.200315

Two New Cases of Pulmonary Infection by *Mycobacterium shigaense*, Japan

Appendix

Chemotherapy Regimen for the 2 Patients

We conducted therapeutic drug monitoring for 2 patients with pulmonary disease caused by *Mycobacterium shigaense*. We suspected a chronic respiratory tract infection and started broad-spectrum antimicrobial drug therapy: amoxicillin/clavulanate (2:1) (375 mg, 3×/d). On day 15, the treatment was changed to tazobactam/piperacillin (4.5 g, 3×/d), which was not effective. On day 26, *M. shigaense* was isolated from a cultured sputum sample. Thereafter, injected amikacin (400 mg/d) and imipenem/cilastatin (0.3 g, 3×/d) and oral clarithromycin (400 mg, 2×/d) were administered. The pulmonary shadow was partially reduced. However, the patient had gastrointestinal adverse effects, including nausea and vomiting. On day 40, imipenem/cilastatin was adjusted according to trough levels and administered 3×/wk), and adverse effects were gradually reduced. Consecutive acid-fast bacillus in sputum samples were smear-positive by Ziehl-Neelsen staining. On day 56, treatment proceeded to a continuation phase of oral clarithromycin with 2 additional antimicrobial drugs (ethambutol and rifampin). After 12 months of daily antimicrobial drug therapy, chest CT showed a decreased extent of bilateral consolidation and no notable side effects.
